# Experiences of the Urban Green Local Environment as a Factor for Well-Being among Adults: An Exploratory Qualitative Study in Southern Sweden

**DOI:** 10.3390/ijerph16142464

**Published:** 2019-07-11

**Authors:** Hanna Weimann, Jonas Björk, Carita Håkansson

**Affiliations:** Division of Occupational and Environmental Medicine, Medicon village, Lund University, SE-223 81 Lund, Sweden

**Keywords:** green area, green space, interviews, public health, urban planning, well-being

## Abstract

The amount and quality of greenness in the local outdoor environment has repeatedly been linked to human well-being. Different types of green areas are likely required in order to meet the various needs of people throughout the course of their lives and with regard to individual social and economic living conditions. The aim of the present study was to increase the understanding of different pathways between green environments, well-being and health. We conducted 16 interviews to explore perceptions and experiences among adults residing in a semi-urban to urban area and derived categories and subcategories from the data using content analysis. We identified six categories; promoting activities, supporting social contacts, stimulating sensory impressions, providing a retreat, offering ways to influence and creating a sense of coherence, and we recognized that the availability to, and contrasts between and maintenance of the environment were experienced as prerequisites for health-promoting properties of the green local environment. The results illustrate a rich variety in potential pathways through which the green local environment may promote well-being. The study highlights the need to plan the local environment from multiple perspectives, as well as carefully considering prerequisites of various kinds in order for the green environment to support health across the life-course.

## 1. Introduction

A large part of people’s lives is spent at home or in the most immediate local outdoor environment and the characteristics of greenness in these areas are likely to influence well-being [1,2]. As indicated in multiple quantitative studies, access to green areas in close proximity to the residence appears to have implications for both mental and physical health [3,4,5,6,7] with varying importance across the life-course and between individuals with different social and economic living conditions [8,9,10]. Also, among people living in deprived urban environments, contact with nature and green areas has been shown to help mitigate stress [11], although with different stress coping strategies depending on current life stage [12]. Positive perceptions of green areas have also been associated with neighborhood satisfaction [13].

Amount, quality and characteristics of green areas can be measured in multiple ways [14,15,16], including purely objective measures such as the proportion of the total land mass consisting of greenness [17] or more subjective measures like perceived sensory dimensions within the environment [18]. The latter study explored what people wish to experience in public parks and urban green areas and identified eight dimensions; serenity, nature, species richness, cultural history, space, refuge, social and prospect. The inherent meaning of these eight dimensions has much in common with the Attention Restoration Theory (ART). ART describes how fatigue and exhaustion, caused by an overload of directed attention, can be reduced by spending time in or watching natural environments [19]. Results from quantitative studies indicate stronger associations with health when subjective, rather than objective, measures of the green environment are used [20,21]. Qualitative methods propose a complement to quantitative studies and have the potential to generate a greater understanding of the health promoting aspects of the green local environment.

Qualitative studies have elaborated on the subject [22,23,24,25,26] using multiple methods, including qualitative measures of park visits [27,28] and favorite places [29,30,31,32]. Studies have also addressed social capital and how environments that favor human interaction may generate health promoting effects [33,34]. Nevertheless, few qualitative studies have more freely explored the health promoting aspects of the urban green local environment surrounding the respondents’ own residences. While most studies have focused on the experiences of specific parts of the population like children [35,36], adolescents [37], older people [38] or vulnerable groups [39,40], it is less common that studies reflect the views of adults in the general population.

Urban planning has often focused on densifying cities, and this approach is likely to threaten both the extent and quality of existing green areas. Such changes in the environment may put the health of the population at risk, as a variety of different types of green areas is needed in order to meet the various needs of children, adults, the elderly or other groups. Consequently, more knowledge about how characteristics of the green local environment are perceived as associated with benefits for well-being may generate hypotheses and ideas for a better and healthier urban planning and development of residential areas. Therefore, the aim of the present study was to increase the understanding of different pathways between green environments, well-being and health by exploring perceptions and experiences among adults residing in a semi-urban to urban area.

## 2. Materials and Methods

A qualitative approach with interviews was used to answer the research question. The data was analyzed using content analysis. This methodological approach allows for in-depth exploration of the experiences of a fairly small number of individuals. Measures to ensure trustworthiness were undertaken following recommendations by Graneheim and Lundman [41]. All subjects gave their informed consent for inclusion before they participated in the study. The study was conducted in accordance with the Declaration of Helsinki, and the study protocol was approved by the Ethical Board in Lund, approval number 2014/863.

Purposive sampling was used to achieve a heterogeneous selection in terms of gender, age and type of housing (i.e., living in an apartment or in a detached building). Individuals fulfilled the selection criteria if they were living in an urban or semi-urban area in Scania, and were between 18 and 70 years of age, able to speak either Swedish or English. The sample included 16 participants, with each participant assigned a study number.

The study was conducted in the Scania region in southern Sweden. The region is characterized by fields and open land in the southwest and larger areas of deciduous and coniferous forests in the northeast. This part of Sweden has clear seasonal variations with spring, summer, autumn and winter, but rarely experiences any major amounts of snow during winter. Scania has a coastline in the west, south and east directions. The participants were predominantly residing in the western parts of Scania, either in any of the two largest cities in the region or in a densely populated village on the countryside.

In order to explore the participants’ experiences of their local environment in general, and the green aspects of this environment in particular, we created a semi-structured interview guide with four main questions. The interview guide was tested in one pilot interview, resulting in only minor adjustments of the guide. In the first question, participants were asked to describe the characteristics of the local green environment surrounding their current residence. They were then asked to explain and describe how the characteristics of the environment affected their well-being, including their physical and mental health, and how they used the environment in their daily life. Finally, the participants were asked to think about places where they had previously lived and to reflect upon if and how the green local environment of the previous places affected their experiences at the current residence. All participants were asked to consider all parts of the year when responding. It has been previously proven that there are wide variations in how people define their local environment [42]. Therefore, the size or shape of the green local environment was not stated in the interview guide. Instead, each participant was free to independently define the boundaries of this area according to his or her own interpretation. Consequently, the areas described by the participants range in distance from the residence, from only reflecting their own garden or balcony, to also include woodlands on driving distance. Only answers reflecting outdoor areas were included in the data analysis. In addition to the descriptions of how the participants used the environment in their daily life and how the environment affected their well-being, we also reflected on possible prerequisites necessary for the green local environment to be perceived as health-promoting by the participants. In order to characterize the study sample, participants also filled out a very short questionnaire about their age, gender, education, weekly physical activity level and type of housing (i.e., living in an apartment or in a detached building).

Each participant completed one interview. The first interview was conducted as a pilot interview in July 2014. The following interviews were conducted between March and April in 2015 or between January and May in 2016. The first author conducted all interviews either in the home or workplace of the participant, or at our research office. Only the interviewer and the participant were present in the room during the interview. Interviews were audio recorded with a voice recorder. The mean duration of the interviews was 43 min (ranging 24–65 min). The research team consisted of the three authors.

All interviews were transcribed by the first author and analyzed using content analysis. Each interview was read multiple times in order to obtain a deeper understanding of the content in relation to the research question. We analyzed the data for meaning units within each separate interview and coded the units. Codes with approximately the same meaning were then combined into groups within each interview. In the next step, we joined the codes from the first interview with the codes of the second interview and combined them into a rough outline of groups. This process was repeated by adding the codes from the next four interviews. The groups were then named and regarded as categories. For each category we also found a varying number of subcategories. The codes from the remaining interviews were added and data was regrouped until the final pattern of categories and subcategories emerged. The first author was in charge of the data analysis and all parts of the analysis was discussed and redrafted in close cooperation with the third author. All three authors were actively involved in critical discussions until consensus regarding the data analysis was reached. The analysis process resulted in six categories which all describe how the participants experienced that the urban green local environment was related to well-being. As an additional result, we identified three prerequisites relevant for the health-promoting potential of the six categories.

## 3. Results

### 3.1. Participant Information

The participants included in the final study sample were between 26 and 70 years of age at the time for the interview (mean age 50 years). Additional characteristics of the 16 participants are presented in Table 1.

### 3.2. Experiences

The data analysis process resulted in six categories which all describe ways in which the participants experienced that the green local environment was related to well-being. For all categories except one, a varying number of subcategories were also identified (Table 2).

#### 3.2.1. Promoting Activities

The green local environment promoted an active life in two ways. The first was through its potential to motivate the participants into being active. The second was through the possibility to discover various places and environments and to make choices on where to spend time. The type of activities promoted by the environment were both physical and social activities, as well as activities like cultivation and gardening.

- Motivation. Participants described that the presence of greenery motivated them to be physically active, especially if these environments were experienced as varied and authentic. They described being drawn to and actively seeking green areas for activities like walks, bicycling trips and runs. Doing gardening work in their own garden was another inspirational and meaningful leisure time activity mentioned by the participants. Being physically active gave the participants a feeling of accomplishment, which in turn motivated even more physical activity. If the physical activity was performed together with a person that the participant enjoyed spending time with, this could create an additional level of value.

*“I have always liked being in nature and being surrounded by greenery. And being able to now go and do that, do these things, and combine training with being outside in nature at my own choosing, not being wheeled around in a wheelchair, but doing something active. That makes me feel good and it is an extra incentive to keep on doing that.”*

*(Participant 6)*

- Fascination to discover. Being able to make new discoveries stimulated participants to explore the green local environment further. Trying new walking paths and visiting new places as well as rediscovering familiar paths and places encouraged the participants to explore their local environments and created added value in life. The possibility to make active choices as to which places and surroundings to discover created a break from the normal environment and made the participants feel more relaxed. The opportunity to make both new and old discoveries was appreciated.

*“It is this that you walk up and down, not really knowing what will happen around the next little hill. And then you’ve also got some paths where you could almost walk blindfolded”*

*(Participant 10)*

#### 3.2.2. Supporting Social Contacts

Features of the green local environment had an impact on its potential to influence the opportunities for social contact with friends and family, as well as neighbours and other people in general. Participants described the open public green areas as places to socialize and dine outdoors and that doing so was beneficial for their social lives and interactions with others. Participants commonly described it as easier to make and keep contact with the neighbours and to maintain good relationships with them when living in a detached building compared to an apartment building, as in the outdoors, the environment made people more visible to each other. In areas with detached buildings, their own garden could also serve as a basis for conversation topics between neighbours.

*“It’s easier to have contact with neighbours in a villa than in an apartment building area. Now, we still have the privilege. Some parts of the yard here are common, so that makes you meet others.”*

*(Participant 10)*

#### 3.2.3. Stimulating Sensory Impressions

Participants expressed that the green local environment influenced the senses and created impressions which affected their health in a positive way. This occurred through visual stimuli, sound experiences, experiences of light and how the environment changes over time. The latter subcategory describes aspects that go beyond the visual senses and provide a deeper understanding of how the environment develops over time and how this is affecting the well-being of the participants.

- Visual stimuli. Beautiful and spacious views resulted in feelings of calm and satisfaction. The view increased the perceived size of the living space or the garden and could also serve as a reward after walking up a hill. Visual stimuli were also provided through experiences of beauty like seeing the different colours in trees, flowers and plants. Watching the flowers of spring and summer and the blended colours of autumn also affected the participants and gave them a feeling of happiness and satisfaction.

*“Especially the parks that are close by, when they begin to bloom, it feels like that, well, but then you really see the park for itself. The flowers and colours have got some kind of effect, at least on me, because otherwise it is this ordinary grey concrete environment.”*

*(Participant 12)*

*“For well-being, I think it is very large indeed. So that. I have lived a few times with a really nice view, and I noticed that I felt very good out of it.”* and *“Then maybe even that. Now I have lived in so many places, but if it’s a bit ugly, what I perceive as ugly views, one might feel a little trapped in a different way. That you want out and away.”*

*(Participant 5)*

- Sound experiences. The sound experiences described by the participants focused mainly on two types of sounds; sounds from birds, wind, waves and running water, and city noise from cars, buses, airplanes and other people. The nature sounds were described as something positive, and the lack of city noise made it easier to experience the sounds of nature. Absence of city noise and noise from other people provided a sense of relief while sounds from water made the participants feel calm.

*“A lot of the experience of nature is also sounds, I guess. And the less you interfere, so to speak, or the less sounds you cause yourself, the more you will experience, somehow, when you are out in the wild. I think in any case. I am out. I photograph quite a lot so then I am out quite a lot by myself, actually. And then I experience very much, because then I can hear things that I would otherwise never hear. I mean, it could be birds flying past. I hear, you hear the wing beats.”*

*(Participant 3)*

- Experiences of light. Participants cherished the sun and described how the awaited return of more sunshine in the spring gave them energy and joy of life. Having access to an outdoor place at home, where you could take a seat and enjoy this experience of light, was described as crucial for well-being. It was important for the participants that the outside space where they lived was light and bright. Too many trees and plants could create shadows and result in a feeling of darkness and lack of impressions.

*“I moved to this house and took it over after my mother a few years ago. And it was a little difficult at first, because it felt a little, like I almost looked out on a wall of greenness. And when it was dark, then it was dark. So it felt a little weird and a little, not insecure, but it was too few impressions, so to speak.”*

*(Participant 7)*

*“In the spring when the sun comes out and you go out and sit and have a coffee in the sun. It is wonderful. Then the spirit comes alive again.”*

*(Participant 9)*

- Change over time. The experience of change was most obviously expressed through the variations occurring during the transitions between spring, summer, autumn and winter. The participants described how experiencing the seasonal variations, like discovering the first flowers of spring or witnessing when the autumn leaves fall from the trees, made them rediscover nature year after year. Their well-being was affected by seasonal changes and participants were happier and more alert during spring and summer. Watching plants and trees grow bigger also gave insight into the changes over time.

*“It is nice just to walk and sit down in the park. And I have been there during so many years. It is always so that you come back to see, how much has that plant been growing and so.”*

*(Participant 10)*

*“One notices in spring and summer, especially in the spring, as it is now, I notice. You sense that you feel much better. Happier, more alert in many ways as well. And then when autumn comes and it like becomes darker. It is sort of, you don’t have as much energy and so on. So you notice that.”*

*(Participant 16)*

#### 3.2.4. Providing a Retreat

Green environments had the potential to offer shield and relief from the demands and pressure that the participants sometimes experienced in life. Green environments also provided a sense of serenity and helped participants to manage thoughts and emotions.

- Freedom from demands. Participants described how spending time in nature or in their own garden reduced the perceived need to perform and provided the participants with a feeling of adequacy. This removed any latent feelings of anxiety and stress and created peacefulness. Participants described how, when being in a green environment, all impressions arose in a slow pace, without any instructions on what to do next.

*“You never sense any stress when you are out in nature. Not me, anyway. You feel, you know, you breathe fresh air and you sort of have no roof over your head and it does not feel hard or that you have to perform. You can just go there basically. And then you look at whatever you want.”*

*(Participant 3)*

- Serenity. The green local environment and nature provided serenity through the presence of beautiful greenness, woodlands and calm waters as well as animals, plants and flowers. These places reinforced the pleasant sedative effects and made participants feel safer and less stressed which, in turn, increased health and well-being. To spend time in environments that the participants were fond of gave them feelings of calmness, safety and happiness. To be out walking in a green environment also made them more relaxed.

*“Overall in the last year since we lived there, my health has gotten better. I have made more progress in my rehabilitation and I think it plays a role where we live because we are quite happy and content there. Not just with the apartment but really with the surroundings that plays a big role for us.”*

*(Participant 6)*

- Emotional management. Feelings could be more easily managed by the participants in green environments compared to other environments. Spending time in, or taking a walk in a park or a forest was described as a good way to clear the mind and let go of experiences that might otherwise be perceived as burdensome. It was also a way to gain energy and helped participants to process negative feelings, especially when having a hard time.

*“I really sense that too. Would I have a tough time for a period, for some reason, I would certainly be out and walk a lot in the woods. And be out in the garden a lot and also out in the woods. I know that it makes me feel good.”*

*(Participant 3)*

#### 3.2.5. Offering Ways to Influence

The well-being of the participants was positively affected if they experienced opportunities to influence their own private environment. This could be done through cultivation and gardening, which was sometimes a way to express creativity. Their own garden or balcony also provided a place for self-determination and autonomy, as this environment could be shaped to suit personal taste and needs.

- Creativity. The participants felt that being able to influence the use, design and appearance of their garden, balcony or other outdoor space close to the home was a way to express creativity and to produce something out of their own minds. They explained how they enjoyed the feeling of planting a tree, plant or flower, and watching it grow. Sometimes the result was as they had expected and sometimes not, and this was part of the creative process. Watching the gardens of other people aroused curiosity and was a source of inspiration. The participants’ thoughts about cultivation were closely related to aspects of creativity, although it included also the positive experiences from growing and harvesting their own food. This was highly appreciated by the participants as it provided them with feelings of joy, energy, strength and an added level to their well-being.

*“To produce your own food, to see when things are growing, it’s very special. I think that it is something that most people can appreciate and feel some sort of power in.”*

*(Participant 7)*

- Self-determination. A feeling of self-determination was achieved through the access of a private environment, like a garden, a part of a garden or a balcony which the participant was free to use and design according to personal preferences. In order for this space to provide feelings of relaxation and refuge and have positive impacts on well-being, it was important that it was easy to access from the indoor space. The size of the area also needed to correspond to the time and energy needed in order to keep the surface in a condition which fulfilled the participants own demands, otherwise it could result in feelings of inadequacy. Having this private green environment made the participants feel safer and was beneficial for their well-being.

*“It is some sort of basic security. This is truly mine. Here is my lawn, here I go out, here I do just as I want. I do not have to consider anyone else. It will be as I shape it.”*

*(Participant 7)*

*“You may just put down some flower bulbs and some plants and then you can water it a little bit and then you can remove some weed sometimes. But you do not have to, you can do as much as you want”* and *“Just that there is a small space, just a little. I really don’t think that you need to have a huge area. And then you may not feel that it is so overwhelming to manage it.”*

*(Participant 11)*

#### 3.2.6. Creating Sense of Coherence

Participants experienced positive emotions related to the feeling of being part of a larger unit. This additional context provided by the green local environment was expressed both through a feeling of coherence with other people using the environment and through a feeling of interconnectedness with nature itself.

- Coherence with other people. Participants talked about seeing other people use and enjoy the public green areas in their local environment and described how this provided them with a sense of coherence. This feeling arose regardless of whether any direct social exchange with other people took place or not. Watching other people strolling around in the park or using the playgrounds created a sense of life and movement in the area and gave the participants feelings of joy and satisfaction.

*“I think it is important that there is some life and movement. And that there are areas that are perceived as pleasant, so that it is used by others, so that people move around there. That it is not only scary bushes or so. I think it does a lot. Yes. Especially when you feel that others think that it is nice and want to use it.”*

*(Participant 1)*

- Interconnectedness with nature. This was described by the participants as a feeling of being part of something greater than oneself which, in turn, created feelings of harmony and security. The interconnectedness with nature was reinforced through the contact with and experience of animals. Birds and squirrels were mentioned as particularly important and the experience of riding a horse through the woods intensified the feeling of being a part of nature. This contact with nature was also a source from which to gather new strength.

*“You feel harmonious and you can fetch new strength and feel a bit as a part of the creation. It sounds pompous, perhaps. But so it is. One feels that one is that little man.”*

*(Participant 14)*

### 3.3. Prerequisites

During the analysis process, three prerequisites necessary for participants to experience the green local environment as health promoting emerged. The prerequisites mentioned by the participants were availability to, contrasts between and maintenance of, the green local environment. For the participants, the extent to which the green local environment was appreciated and used was highly determined by the availability of these areas. Participants highlighted the importance of having easy access to public parks, lawns and forests as well as areas with restricted traffic, as traffic was regarded as limiting the freedom to be active. The occurrence of safe bicycle paths favoured participants’ willingness to use the bicycle for active transport. Having access to open public green areas, preferably with a lot of benches, created possibilities for large groups of people to come together and was important for spontaneous physical and social activities. The likelihood of social encounters with other people increased if stimulating and varied playgrounds and courtyards were available. In general, greenness in the shape of trees and bushes surrounding their own home was described as a protection against the views of other people, which could otherwise make the participants feel exposed. Having lawns between apartment buildings created a positive physical distance to the neighbours, but on the other hand, large housing plots and high hedges could also serve as a barrier between people.

Another prerequisite necessary for participants to experience the green local environment as health promoting was the presence of contrasts. Contrasts in the green local environment were created through the availability of different types of green areas as well as between the green and the built environments. Participants described experiencing positive feelings when they went from an urban area to a greener environment and that the same difference could occur also between greener and less green places in a city. The pure existence of a park or other green space, without the participant actually spending any time there, could be enough for these positive feelings to emerge.

Participants raised concerns regarding the maintenance and design of public spaces. An environment that was experienced as being taken good care of was described as cosy, homely and nice, but if the maintenance was not perceived to be good enough, this could cause feelings of anxiety. Participants expressed fear towards the possibility that parks near their home would be built upon. These concerns did not only regard themselves, but also other people, especially families with children. The struggle for preserving green areas brought commitment among participants. Participants also felt that too much space in the cities was paved with stone when it could have been greener, and this raised concerns about the health of future generations.

## 4. Discussion

The aim of the present study was to increase the understanding of different pathways between green environments, well-being and health, by exploring perceptions and experiences among adults residing in a semi-urban to urban area. We identified six categories which respond to the aim and also found three aspects that were prerequisites for the health promoting properties of the green local environment. The categories were; promoting activities and supporting social contacts which evolve around physical and social activities; stimulating sensory impressions which describes phenomenon that the participants experienced through their senses; providing a retreat and offering ways to influence which define aspects provided by the environment that has psychological, rather than physical, properties; and creating sense of coherence which describes the importance of being part of a larger context.

The Attention Restoration Theory (ART) was presented in the mid-1990s and proposed an original rationale for the restorative properties of natural environments. The ART describes how fatigue and exhaustion, caused by an overload of directed attention, can be reduced by spending time in or watching natural environments [19]. Parts of our results clearly support the ART, especially the providing a retreat category which demonstrates the inherent power of nature to provide serenity and a place where the participants could rest and feel free from the demands of everyday life. But also the stimulating sensory impressions category, in which beautiful and spacious views gave feelings of calmness and satisfaction and the sounds of nature provided a sense of relief, are in line with the ART. Serenity, nature, species richness, cultural history, space, prospect, refuge and social are eight dimensions that describe what people wish to experience in public parks and urban green areas and was identified around the same time period as the ART was presented [18]. Providing a retreat category has many similarities with the dimension’s serenity and space, but also with refuge that has, together with the dimension nature, been shown to be correlated with stress and a need for restorative environments [43]. Offering ways to influence category has similar properties, but not the exact same as described in the providing a retreat category. Although most of our participants had access to a private green area of some kind, this resource is far from accessible to everyone. To provide increased possibilities for public gardening and other ways to influence and be creative is an important task within local urban planning. However, these public resources must be characterized by good organization and provide space and capacity for privacy in order to fulfil the properties of the offering way to influence category.

The experiences described in the supporting social contacts category are similar to the properties of the social dimension, and to provide the green local environment with attractive green areas and meeting places has been suggested as interventions to increase human interactions and social capital [34]. A less noted but very interesting aspect of the green local environment was its ability to create a sense of coherence, both with nature itself and with other people. As described by the participants, the coherence with other people arose from seeing the public green areas being used and enjoyed and did not require any physical contact with other people. This differentiates the subcategory from the supporting social contacts category, which evolves around socializing activities with friends, family, neighbours or others, but it still has properties similar to the social dimension.

The association between characteristics of the green local environment and physical activity [44] or social contacts [34,45,46] has been examined in multiple studies and these factors were also mentioned by the participants in our study. In the promoting activities category participants described being drawn to and actively seeking green areas for activities like walks, bicycling trips and runs, and how the possibility to make new discoveries stimulated them to explore the green local environment further. Physical activity was often combined with social activities, either because the participant conducted the physical activity together with another person, or because social activities where people would meet to socialize also encouraged spontaneous sport games and other physical activities.

Both the physical and the social activities mentioned by the participants require access to green local environments with certain properties. A strong prerequisite for the health promoting properties of the green local environment among the participants was availability, which to a high degree determined how and how often the environment was used. This is previously known from quantitative studies [47,48] and was confirmed in our data. The presence of contrasts in the green local environment was a less renowned, but often mentioned prerequisite raised by the participants. The need for contrasts most likely describes a need for multiple impressions and might potentially be viewed as an urban version of the dimension of species richness. The uncertainty about how the local environment would change in the future affected the health promoting properties of the green local environment; so did the fear that highly appreciated green areas would be built upon. These are both negative factors that have been rarely raised in previous research.

The trustworthiness of a qualitative study is evaluated in relation to the methods used to generate the presented results [41]. Data was collected over two periods of time (i.e., from March to April in 2015 and from January to May in 2016), but as the questions were constructed to cover the full year, we argue that this potential issue has been handled. The risk for inconsistency during data collection cannot be ruled out as the interviewer (i.e., the first author) acquired new insights into the research topic and improved her interview skills over time. Also, the previous knowledge on and preunderstanding of the potential health promoting aspects of the urban green local environment of the first author may have influenced the interview process. However, this probably did not affect the rest of the research team or the analysis process. Both the first and the third author read all 16 interviews and agreed on the selection of meaning units. The first author was in charge of the data analysis, but a major part of the analysis process was spent on discussing and redrafting the results in close cooperation with the third author. During the process of writing the paper, the second author also made major contributions to the presentation of the results, which resulted in some additional changes. The close cooperation within the research team increased the credibility of the results.

The results of this study have implications for the design and overall planning of cities and urban areas. Not only the characteristics of public areas like parks etcetera, but also the characteristics of more semi-public green areas like courtyards and allotment gardens seem to be of importance for personal well-being. Many of the participants expressed a fear that their immediate environment could be built upon, and the mere knowledge that this could potentially happen affected their well-being negatively. Furthermore, the uncertainty over if, how and when this would actually become a reality also created concerns among the participants. This illustrates the importance of clear and secure information from the authorities in planning for new construction projects and other changes to the local environment. These concerns and the clear impact on well-being described by the participants in our study demonstrates the extensive health values that are at risk when green areas are being downgraded and regarded as less important. It also shows how important it is to be given the opportunity to influence and discuss the future development of your own local environment, even when no plans to change the current environment have been presented.

As described in the six categories identified in the present study, green areas do not only have the power to support physical and social activities, but has also have a range of psychological, rather than physical, properties. The characteristics of the green local environment have direct as well as more indirect implications on the well-being of the population and a variety of different types of green areas are needed in order to meet the various needs of different groups and individuals. Future research should aim to highlight the value of using qualitative methods as well as mixed-methods approaches as an important complement to quantitative studies. Quantitative studies could attempt to describe the relative importance of the experienced curative aspects and examine the transferability between similar results from qualitative and quantitative studies. Future qualitative studies should also focus more on describing experiences among adults in the general population alongside studies in more specific groups like children or the elderly. The present study identified three prerequisites for the health promoting properties of the green local environment and both the need for contrasts and maintenance of the environment are aspects that require further attention in future qualitative, as well as quantitative, studies.

## 5. Conclusions

The results of this study illustrate the rich variety in potential pathways through which the green local environment may promote well-being. A variety of different types of green areas is likely to be required in order to meet the various needs of different groups and individuals. Our study highlights the need to plan the local environment from multiple perspectives, as well as carefully considering prerequisites of various kinds, in order for the green environment to support health across the life-course.

## Figures and Tables

**Table 1:** Characteristics of the 16 participants.

Characteristics	*n*	%
Gender		
Male	8	50
Female	8	50
Age group		
18–34 years	4	25
35–49 years	4	25
50–64 years	3	19
65–70 years	5	31
Type of housing		
Detached building	9	56
Apartment	7	44
Education		
Primary /sec. school	1	6
University < 3 years	3	19
University ≥ 3 years	12	75
Physical activity per week		
≤ 1 h	1	6
1–3 h	4	25
3–5 h	5	31
≥ 5 h	6	38

**Table 2:** Presentation of the six categories with corresponding subcategories (when applicable).

Categories	Subcategories
1. Promoting activities	Motivation
	Fascination to discover
2. Supporting social contacts	
3. Stimulating sensory impressions	Visual stimuli
	Sound experiences
	Experiences of light
	Change over time
4. Providing a retreat	Freedom from demands
	Serenity
	Emotional management
5. Offering ways to influence	Creativity
	Self-determination
6. Creating sense of coherence	Coherence with other people
	Interconnectedness with nature
